# Gadolinium in the Environment: A Double-Edged Sword for Plant Growth and Ecosystem Stability

**DOI:** 10.3390/metabo15060415

**Published:** 2025-06-19

**Authors:** Marlena Tomczuk, Beata Godlewska-Żyłkiewicz, Andrzej Bajguz

**Affiliations:** 1 Department of Biology and Plant Ecology, Faculty of Biology, University of Bialystok, Konstantego Ciołkowskiego 1J, 15-245 Bialystok, Poland; m.tomczuk@uwb.edu.pl; 2 Doctoral School of Exact and Natural Sciences, University of Bialystok, Konstantego Ciołkowskiego 1K, 15-245 Bialystok, Poland; 3 Department of Analytical and Inorganic Chemistry, Faculty of Chemistry, University of Bialystok, Konstantego Ciołkowskiego 1K, 15-245 Bialystok, Poland; bgodlew@uwb.edu.pl

**Keywords:** bioaccumulation, hormetic effect, gadolinium-based contrast agents, plant growth, rare earth element, toxicity

## Abstract

Gadolinium, a rare earth element, is increasingly released into the environment due to its widespread applications in medical imaging, industry, and agriculture. This review explores the dual role of gadolinium in plant systems, highlighting its potential benefits at subtoxic concentrations and detrimental effects at higher levels. At subtoxic doses, gadolinium can enhance plant growth, metabolism, and stress tolerance by promoting enzymatic activity and nutrient absorption. However, elevated concentrations induce oxidative stress, disrupt nutrient uptake, and impair photosynthesis, leading to cellular damage and reduced growth. The bioaccumulation of gadolinium in plant tissues raises concerns about its trophic transfer within food chains and its broader ecological impact. Current evidence suggests that previously regarded as stable and inert gadolinium complexes can degrade under environmental conditions, increasing their bioavailability and toxicity. Despite its potential for agricultural applications, including improving crop resilience, the ecological risks associated with gadolinium remain poorly understood. Addressing these risks requires coordinated efforts to optimize gadolinium usage, develop advanced waste management strategies, and enhance monitoring of its environmental presence. This review emphasizes the need for in-depth research on gadolinium interactions with plants and ecosystems to balance its industrial benefits with environmental sustainability.

## 1. Introduction

Gadolinium is a rare earth element (REE) widely used in various fields, including medical imaging, manufacturing, and electronics. Its increasing release into the environment, primarily from industrial and medical sources, has raised concerns about its impact on ecosystems, particularly plants. The interactions between plants and gadolinium are complex and depend on its concentration, chemical form, plant species, and environmental conditions. Although research on the effects of gadolinium on plants remains limited, existing studies indicate that, like other heavy metals and REEs, gadolinium can have both beneficial and harmful effects [1,2].

Understanding the effects of gadolinium on plants is crucial for assessing ecological risks and developing strategies to mitigate its adverse effects. Further studies are essential to uncover the mechanisms underlying gadolinium–plant interactions. Such research could focus on identifying gadolinium-tolerant plant species or optimizing phytoremediation techniques that use plants to remove gadolinium from contaminated environments. These efforts are crucial to addressing the environmental challenges posed by gadolinium while exploring its potential applications. The dual effects of gadolinium on plants underscore its concentration-dependent behavior. At subtoxic concentrations, gadolinium has been observed to stimulate plant growth and development by enhancing seed germination and promoting root and shoot growth in certain species. These positive effects may arise from the role of gadolinium in modulating physiological and biochemical processes, such as enzyme activity and nutrient uptake. Exploring these beneficial interactions highlights the potential of gadolinium to enhance plant growth under specific conditions. However, at higher concentrations, gadolinium exhibits toxicity, leading to reduced growth, chlorosis (yellowing of leaves), necrosis (death of tissue), and disruptions in water and nutrient absorption [3,4].

Gadolinium toxicity also induces oxidative stress, characterized by the overproduction of reactive oxygen species (ROS) that damage cellular components such as DNA, proteins, and lipids. The mechanisms underlying gadolinium toxicity are not fully understood, but it is believed that gadolinium disrupts cellular processes by competing with essential cations like calcium and magnesium, impairing photosynthesis, respiration, and osmotic balance [5]. This dichotomy emphasizes the importance of understanding effects of gadolinium across varying concentrations. While it may enhance plant growth under controlled conditions, its toxic effects in contaminated environments pose significant challenges. Further research is imperative to elucidate these interactions, mitigate adverse effects, and potentially exploit the beneficial properties of gadolinium [2,6].

This review delves into the multifaceted role of gadolinium in plant systems, exploring its dual effects as a growth stimulant at subtoxic concentrations and as a toxic agent at higher levels. The discussion explores the ability of gadolinium to enhance physiological and biochemical processes, such as nutrient uptake and enzyme activity, and also addresses its adverse impacts, including oxidative stress and nutrient competition. Special attention is paid to the fate of gadolinium in the environment, including its sources, pathways, and bioaccumulation potential, as well as its interactions with various plant species. This review synthesizes current knowledge to provide a comprehensive understanding of both the risks related to gadolinium and its potential benefits in plant systems. It also identifies research gaps and proposes directions for future studies.

## 2. Gadolinium in the Environment

Gadolinium occurs in the environment primarily in various minerals, such as bastnasite, monazite, loparite and laterite clays, along with other REEs. The common presence of gadolinium together with other REEs results from similar chemical properties and affects the behavior of individual elements in the environment [7]. The ability of Gd to substitute other REEs can lead to competition for sorption sites and organic ligands, which affects their mutual mobility [8]. In the aquatic environment, the chemical form of REEs strongly affects their availability and toxicity, and some studies indicate the additive toxicity of individual elements—a synergistic effect [9]. In the case of plants, there are studies that have shown that monocellular organisms may not be able to properly distinguish REEs and have the same toxic effect both when treated with a mixture of REEs and with a single element [2].

Additionally, human activity contributes to its presence in the form of metal complexes with various chelating ligands. Most gadolinium compounds are poorly soluble in water; however, under certain conditions, they can form more soluble salts that dissociate in water. While ionic gadolinium (Gd^3+^) is highly toxic, its high reactivity means that it usually exists in a bound form in the natural environment [10].

The Gd^3+^ is known to mimic essential cations, such as calcium (II), zinc (II), magnesium (II), and iron (III), which may act as cofactors in numerous cellular and biochemical processes [11,12]. This displacement is caused by comparable atomic radii and is the reason for the toxic effect of gadolinium [7,13]. Gadolinium exhibits a strong affinity for organic molecules containing oxygen, nitrogen, or sulfur, which serve as electron donors [3]. Imaging and diagnostics utilize the unique magnetic and spectroscopic properties of lanthanides. Due to its particularly high magnetic moment resulting from seven unpaired 4f electrons and a long electronic relaxation time, Gd^3+^ enhances the longitudinal relaxation rate of the ^1^H nuclei of water surrounding tissues of interest, enabling high-resolution imaging of internal body structures [7].

**Figure 1:**
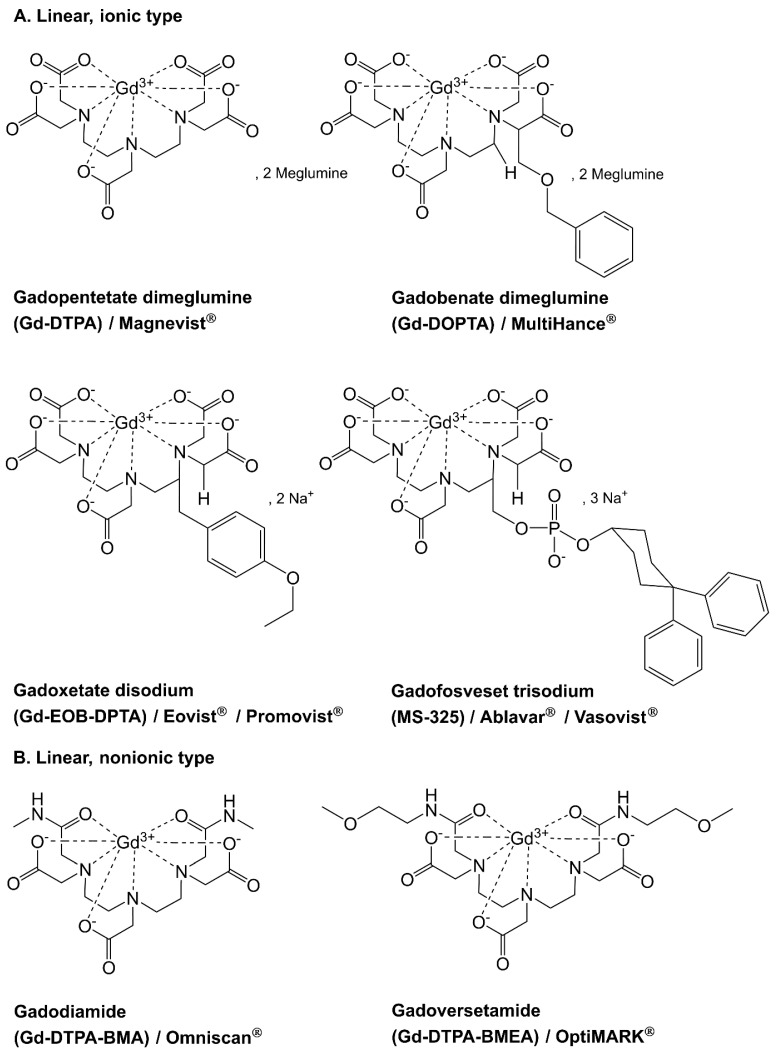

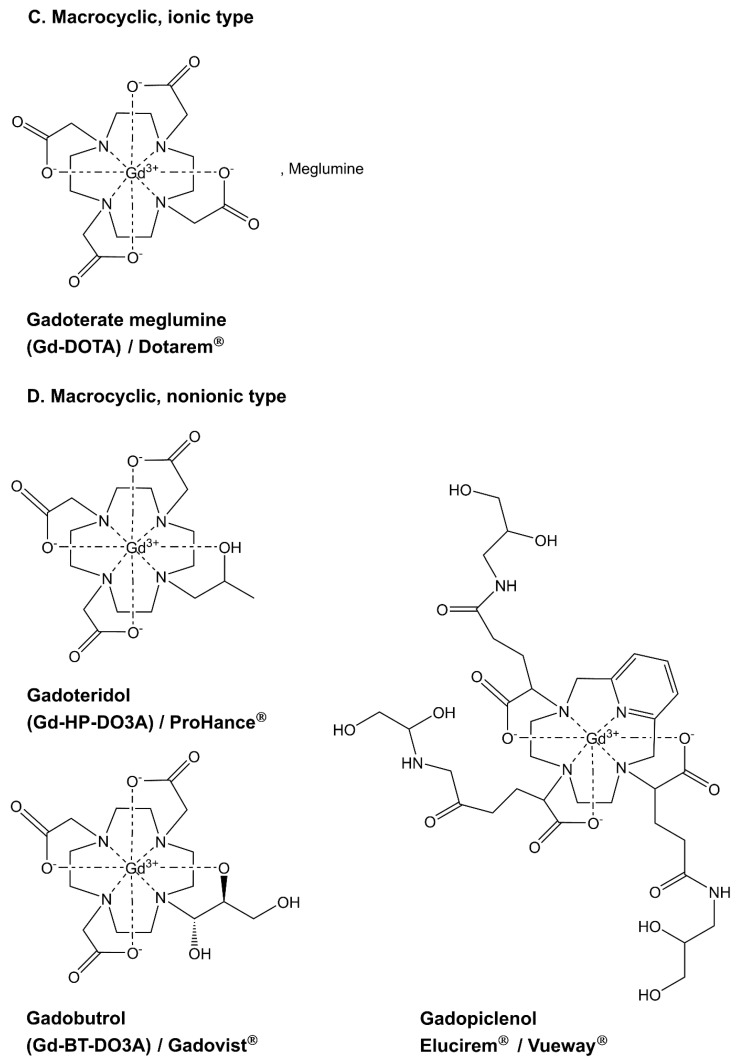
Structural formulas of gadolinium-based contrast agents with trade names used in medicine.

Gadolinium-based contrast agents (GBCAs) available on the market differ in their chemical structure, linear (Figure 1A,B) or macrocyclic (Figure 1C,D), and the overall charge of the compound, which determines their stability and imaging properties [14]. Macrocyclic compounds exhibit increased stability due to electrostatic interactions between the gadolinium ion and ligands forming five-membered rings, resulting in the formation of a molecular cage that reduces the likelihood of dissociation [15,16,17]. Numerous studies have investigated the kinetic behavior of linear and macrocyclic complexes [18,19,20]. It is generally accepted that gadolinium-based compounds with lower kinetic stability and higher dissociation rates pose a greater risk of releasing free gadolinium from the chelate structure [15]. The dissociation of linear complexes accelerates under acidic conditions and at elevated temperatures in comparison to macrocyclic contrast agents [14]. These findings highlight the importance of further research on the environmental impact of these compounds, particularly under uncontrolled conditions [21]. Recently, several gadolinium–colloidal structures have been investigated as contrast agents, and the possibility of using inorganic nanoparticles (e.g., Gd_2_O_3_, GdPO_4_, NaGdF_4_, GdF_3_) for this purpose has also been assessed [22,23]. To date, there are no studies on the fate of such compounds in the environment.

### 2.1. Sources of Contamination and Fate of Gadolinium in Soil and Water

Gadolinium is released into the environment both through natural processes, such as rock weathering, and through human activity. Gadolinium has a wide range of industrial applications, including use in lasers, magnets, hard drives, monitor screens, metal alloys, glass additives, and control rods for nuclear reactors [24]. Its ability to capture neutrons makes it an ideal material for regulating activity in reactor cores. Furthermore, the addition of gadolinium oxide to uranium oxide pellets improves fuel burnup efficiency in nuclear power plants. In neutron radiography, gadolinium is used to produce high-resolution images while preserving sample integrity [25].

**Figure 2:**
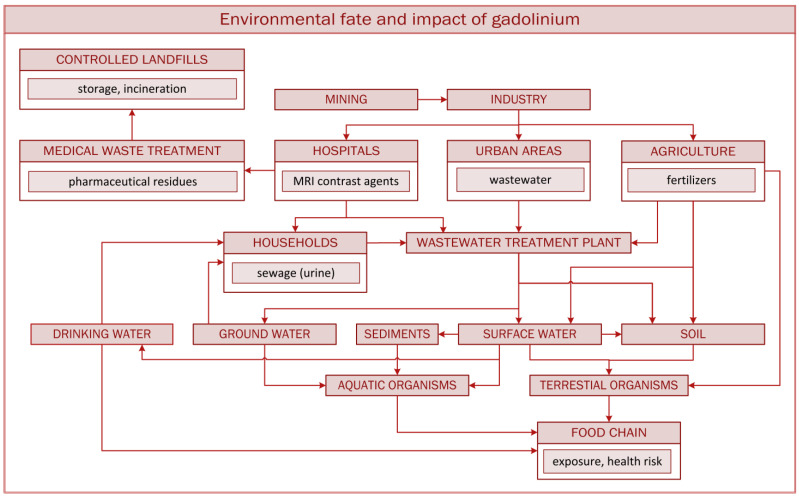
Gadolinium circulation in the environment.

The largest source of anthropogenic gadolinium (Figure 2), however, is its use in magnetic resonance imaging (MRI) and magnetic resonance angiography (MRA) as GBCAs [26,27]. After administration to patients, these compounds are excreted in urine, directly entering municipal sewage systems. Industrial and hospital discharges further contribute to gadolinium contamination.

Hospitals dispose of unused contrast agents together with other pharmaceuticals, such as antibiotics, anti-inflammatory drugs, and antihistamines, contributing to the emergence of an anthropogenic gadolinium anomaly [28,29,30]. It has been estimated that, on average, about 11% of GBCA is disposed of and lost after each MRI examination [31]. Medical residues are collected in special containers and typically sent to licensed hazardous waste disposal facilities. These facilities use methods such as incineration or chemical treatment to neutralize or safely dispose of the substances [32].

Wastewater containing gadolinium is subjected to multi-stage purification processes in a sewage treatment plant, but the removal efficiency is only 5.0–37% [33]. This means that approximately 63–95% of gadolinium compounds remain in treated water, eventually entering surface waters (rivers, and lakes), infiltrating soil and groundwater, and ultimately contributing to the contamination of drinking water sources and accumulating in aquatic and terrestrial organisms [13,30,34]. These compounds persist in the environment due to their high stability and long half-life. Moreover, during the treatment procedures in a WWTP, transformation and/or dissociation of gadolinium chelates may occur, which may lead to the release of free Gd^3+^ ions and other gadolinium species [13]. Chemical reactions can alter the chemical forms of gadolinium, impacting its mobility [17]. For example, Möller and Dulski [35] reported that about 10% of gadolinium in complexes with other rare earth elements undergoes transmetallation. Additionally, other studies have detected unidentified gadolinium complexes in water samples [13,35,36,37]. Contrary to earlier assumptions, some studies have shown that gadolinium compounds can be degraded by UV exposure. UV treatment facilitates gadolinium degradation, potentially releasing free ions into the environment, which may then form complexes with other compounds [38]. Environmental factors such as sunlight, salinity, temperature, and pH influence the stability of GBCAs. Guerreiro and Brito [21] studied the stability of the gadoterate meglumine (Gd–DOTA) complex, one of the most stable GBCAs, under various conditions. They found that the compound remained stable in high temperatures and environments ranging from slightly acidic to alkaline. However, under sunlight exposure, a slight increase in free gadolinium occurred at pH below 4. Additionally, Birka et al. (2016) [39] emonstrated that gadolinium compounds such as gadopentetate dimeglumine (Gd–DTPA), Gd–DOTA, and gadobutrol (Gd–BT–DO3A) remain stable under UV radiation, although their degradation is slowed in surface water matrices.

In addition, gadolinium may enter soil and groundwater through agricultural use of mineral (e.g., Changle and Nongle) or organic (sewage sludge) fertilizers or the disposal of waste in landfills (Figure 2) [10,24,40,41]. REEs, including gadolinium, are applied as foliar biostimulants. Field experiments with rice, wheat, soybeans, peanuts, tobacco, and other plants have confirmed the positive effect of REE mixtures on plant growth [7,42]. Gadolinium and other REEs can interact in soil with inorganic ligands, forming poorly soluble fluorides, carbonates, phosphates, and hydroxides. Reactions between gadolinium and humic acid, the major component of organic matter in soils and water, may also occur. Due to its numerous functional groups and polymeric structure, humic acid forms strong coordination bonds with gadolinium, especially with linear GBCAs (gadodiamide), which reduces its toxicity [18]. These complexes limit gadolinium bioavailability for plants and contribute to environmental stabilization by binding free ions. On the other hand, such complexes can lead to gadolinium accumulation in the environment [43].

### 2.2. Current Levels of Environmental Gadolinium and Trends

Gadolinium exhibits high natural variability of up to 2–3 orders of magnitude depending on the type of environment. The natural geochemical background is the basis for the correct assessment of potential anthropogenic contamination. In unpolluted European environments, gadolinium concentrations range from 0.2 mg/kg to 36 mg/kg in topsoil and <0.1 mg/kg to 16 mg/kg in subsoil, depending primarily on the composition of the parent rocks, texture, weathering history and pyogenic processes, organic matter content and reactivity, pH, and cation exchange capacity of the soil [10,44]. In other natural compartments, Gd levels range from <0.002 µg/L to 0.97 µg/L in stream water, while stream sediments contain between 0.2 and 90.5 mg/kg and floodplain sediments range from 0.21 mg/kg to 22.6 mg/kg with elevated values near urban areas reflecting anthropogenic anomalies [45].

In 1996, significant gadolinium anomalies were first reported in German rivers, marking the beginning of systematic studies on the environmental impact of this element [46]. This discovery highlighted the presence of gadolinium as a trace contaminant in aquatic environments, primarily attributed to its widespread use in medical imaging as a component of contrast agents. These results prompted extensive research into the sources, pathways, and ecological effects of gadolinium, establishing it as an emerging environmental contaminant. Since then, gadolinium concentrations have been steadily increasing in various regions worldwide. A 20-year study conducted in San Francisco Bay confirmed that the accumulation of gadolinium is a reliable indicator of environmental pollution [47]. Elevated gadolinium levels have been detected in surface water in Europe, the United States, Japan, Brazil, China, and Australia, with the highest recorded concentration of 184 ng/L in Germany, compared to a natural background level of approximately 0.63 ng/L [46].

In recent years, there has been a marked increase in gadolinium concentrations in surface and groundwater compared to the geochemical background, especially in urban and industrial areas, due to its extensive use in medicine and industry [24,48]. Currently, thousands of liters of contrast agents are used annually, driven by the growing number of MRI procedures and technological advancements. It is estimated that approximately 150–180 million doses of GBCAs are administered worldwide each year. Based on an average dose of 1.20 g of gadolinium per patient, this equates to 180–220 tons of gadolinium annually, representing about 5% of global production [30,49,50,51]. In Germany, 132 kg of gadolinium was used in 2009, with concentrations in hospital wastewater ranging from 8.50 to 30.1 μg/L [30].

Environmental gadolinium concentrations range from ng/L to mg/L, influenced by factors such as urbanization, the frequency of MRI examinations in a given area, and proximity to anthropogenic sources [52]. Bau and Dulski [46] reported that the gadolinium concentration in some river areas after wastewater discharge ranged from 0.2 to 1.1 mg/L. Subsequent studies confirmed the presence of gadolinium in surface waters and sediments, with concentrations reaching up to 1100 ng/L and 90.5 mg/g, respectively [1]. Toxic concentrations have been recorded for the contrast agent Gadovist at 3.63 µg/L, which is higher compared to other GBCAs [30]. Interestingly, gadolinium concentrations in hospital effluents vary throughout the day, with lower levels observed on weekends due to reduced MRI activity [53].

The widespread emission of gadolinium into the environment is evident from studies detecting measurable concentrations in tap water and even in soft drinks served at fast-food chains in six major German cities [54]. Gadolinium has also been detected in drinking water in Berlin, London, and Prague [55]. This highlights gaps in pollution control measures and suggests that the presence of gadolinium in drinking water is becoming increasingly common. Once gadolinium enters rivers, lakes, and other water bodies, it affects the functioning of entire ecosystems. Although most studies focus on gadolinium concentrations in water, limited studies have been conducted on its accumulation in plants. It is known that due to natural gadolinium emissions, its content in root vegetables is below 2 mg/kg of dry mass, which, compared to the natural content in soil (0.1–16 mg/kg), indicates low mobility of the element in typical soil conditions. However, certain plant species, such as lichens and ferns, can accumulate up to 500 mg/kg of dry mass [1]. Ferns, such as *Phytolacca americana,* classified as REE hyperaccumulating plants can accumulate up to 1500 mg/kg of REE in roots and 100–280 mg/kg in leaves. Notably, *Dicranopteris linearis* can accumulate as much as 0.3% in necrotic lesions of the leaves [56,57].

## 3. Gadolinium Uptake by Plants

The accumulation of gadolinium in aquatic organisms and terrestrial plants contributes to changes in ecosystems, as the element becomes incorporated into tissues. Gadolinium absorption by plants is influenced by various factors, including plant species, the chemical structure of gadolinium compounds, soil properties, the physiological state of the plant, and interactions with other elements. Although the precise mechanism of gadolinium uptake is not fully understood, it is believed to resemble the absorption mechanisms of other metal ions [24].

Research indicates that gadolinium accumulates in different parts of plants at varying concentrations [58]. It has been observed in the cytoplasm, and compared to other lanthanides, it preferentially accumulates in chloroplasts [59]. A significantly larger proportion of gadolinium is found in the underground parts of plants than in above-ground tissues such as stems and leaves [24,60]. Gadolinium ions are absorbed by roots and transported to aerial parts of the plant via the vascular system [58,61].

Due to its ionic radius being similar to that of calcium, Gd^3+^ can be transported by proteins responsible for divalent element transport and, to a lesser extent, through passive absorption [62]. Transport from the roots to aerial tissues occurs via tracheids and vessels, preceded by radial transport through the rhizodermis and endodermal tissues. This process involves Casparian strips and requires gadolinium to form complexes with specific ligands before reaching the xylem [24,63]. Binding with specific oxygen or nitrogen-containing transporters enables the movement of gadolinium through the cytosol to vascular cells, mediated by P-type ATPases [63].

Studies have also examined gadolinium accumulation in plants such as *Lemna minor*, *Lepidium sativum*, and *Zygnema* sp. exposed to contrast agents like gadopentetic acid and gadodiamide [61]. Results showed that gadolinium could be absorbed through roots and, in some cases, leaves. However, for *Lepidium sativum*, absorption occurred exclusively via roots, and no evidence was found of external absorption through leaves. The highest gadolinium concentrations were observed in the main vein of leaves.

The transport of gadolinium in plants is associated with organic acids, including malic acid, citric acid, formic acid, lactic acid, and succinic acid [58]. Among these, malic, citric, and succinic acids play significant roles in gadolinium accumulation in tomato roots and its transport through xylem vessels. Similarly, formic, lactic, citric, and succinic acids influenced its accumulation in leaves. This relationship likely reflects the plant’s adaptation to environmental conditions. Organic acids may also play a protective role by forming complexes with reactive Gd^3+^ in the cytosol, preventing them from interacting with sensitive cellular components. Further studies confirm that gadolinium uptake depends on multiple factors [63]. In experiments on *Chlamydomonas reinhardtii*, gadolinium did not compete with aluminum and iron for uptake. However, its uptake decreased in the presence of other trivalent elements, such as europium, neodymium, thulium, and yttrium [62].

## 4. Effects of Gadolinium on Plants

Research on GBCAs has primarily focused on their effects on human health, with limited attention to their influence on plants. While numerous studies explore accumulation of gadolinium in aquatic ecosystems, its biochemical effects on plants remain underexplored. Available evidence suggests that gadolinium may exhibit effects similar to other REEs, showing a hormetic response: subtoxic concentrations promote plant growth, whereas higher levels disrupt physiological processes [3,10].

### 4.1. Positive Impact on Plants

Studies indicate that subtoxic concentrations of REEs, including gadolinium, can enhance plant growth and development [64,65,66,67]. However, the effects of gadolinium are less understood due to limited research. Growth intensity varies depending on factors such as gadolinium type, concentration, compound form, exposure duration, plant species, and soil conditions [24,41]. Importantly, only bioavailable fractions of gadolinium are absorbed by plants, while total metal content may not directly correlate with uptake [68].

Evidence suggests that gadolinium may stimulate the production of plant hormones and antioxidant enzymes, improve photosynthetic efficiency, enhance nutrient uptake, and support metabolic processes [69,70]. In *Arabidopsis thaliana*, 1.57 mg/L of gadolinium increased root length and biomass [60]. Additionally, optimal growth for *Phaeodactylum tricornutum* occurred at 4.52 mg/L Gd_2_O_3_ [71].

In summary, at subtoxic concentrations, gadolinium can stimulate plant growth by inducing mild stress responses that enhance overall plant performance. Healthy plants exhibit robust growth characterized by green, well-developed leaves, strong shoots and extensive root systems, and well-formed flowers and fruits. Plants may also employ morphological adaptations to minimize damage from metal exposure. Mild stress responses can activate pathways that improve photosynthetic efficiency, nutrient uptake, and antioxidant enzyme activity, promoting better growth and development. Gadolinium has the potential to enhance plant growth and secondary metabolite production, offering promising applications in agriculture [1,64,72,73]. However, further research is necessary to establish safe, non-toxic concentrations and to fully understand its mechanisms of action. This will enable the effective use of gadolinium in agricultural systems.

### 4.2. Negative Impact on Plants

Higher concentrations of gadolinium are toxic, impairing plant growth and development [63,73], and cause oxidative stress, inhibit plant growth, and damage cellular structures. For example, in *Arabidopsis thaliana*, growth was inhibited at 68.7 mg/L, with side effects reported in tomato plants at concentrations exceeding 157 mg/L [58]. On the other hand, *Stevia rebaudiana* showed toxicity at concentrations above 60.5 mg/L, with negative impacts on chlorophyll and carotenoid levels [74]. Algae exposed to gadolinium oxide particles experienced agglomeration, limiting nutrient access and reducing growth [75].

Gadolinium exposure negatively affects plant growth metrics, including root and shoot length, biomass, and reproductive capacity. In *Stevia rebaudiana*, exposure to 605–1814 mg/L gadolinium reduced shoot and root length by up to 80% and 86%, respectively, while biomass decreased by over 40% [74]. Maize treated with 10 mg/L Gd(NO_3_)_3_ showed a 67% reduction in shoot biomass and a 35% reduction in root biomass [41]. In rice, gadolinium concentration of 157 mg/L delayed growth, reducing root length by 75% and shoot length by 58% [73].

Gadolinium exposure also alters the concentrations of essential nutrients, including calcium, magnesium, potassium, and iron. At concentrations above 605 mg/L, gadolinium significantly reduced copper content to undetectable levels, while zinc, calcium, and potassium concentrations also declined [73,74]. In maize, root analyses revealed altered levels of manganese and magnesium, while rice exhibited reductions in sodium and zinc content at toxic gadolinium concentrations. Gadolinium can also replace calcium in cellular enzymes, altering their structure and function. This inhibits antioxidant enzymes and enzymes involved in nitrogen metabolism, photosynthesis, and respiration [3,63].

Gadolinium also affects photosynthetic pigments, i.e., chlorophyll *a* and *b* levels decreased significantly at concentrations above 605 mg/L, with carotenoid levels (e.g., lutein, and zeaxanthin) similarly reduced [74]. Toxic gadolinium concentrations disrupt cellular processes by inducing oxidative stress, characterized by the overproduction of ROS such as superoxide anions (O_2_^•−^), hydrogen peroxide (H_2_O_2_), and hydroxyl radicals (OH^•^). Increased ROS levels exacerbated these effects, impairing photosynthesis and metabolic activity [71]. These ROS degrade proteins, lipids, and DNA, damage membranes, and impair selective permeability [10].

Toxic gadolinium concentrations affect DNA methylation, altering cell cycle regulation and stress responses. In *Arabidopsis thaliana*, gadolinium induced demethylation at 1.57–7.86 mg/L, leading to impaired growth and increased oxidative damage [60]. Some studies suggest that atmospheric CO₂ levels may help mitigate the toxic effects of gadolinium by improving antioxidant capacity, enhancing carbon fixation, and stimulating antioxidant synthesis in *Medicago* species [76].

The toxicity of gadolinium compounds, expressed as EC_50_ values, varies significantly across different plant species and compound types (Table 1).

Free Gd^3+^ exhibits high toxicity, with EC_50_ values as low as 2.2 mg/L for *Pseudokirchneriella subcapitata* and 12.4 mg/L for *Lemna gibba*. As such, gadolinium chloride (GdCl_3_) and nitrate (Gd(NO_3_)_3_) also show considerable toxicity, with EC_50_ values ranging from 1.21 mg/L for *Raphidocelis subcapitata* to 10.2 mg/L for *Skeletonema costatum*. In contrast, GBCAs, e.g., gadopentetate dimeglumine (Gd–DTPA) and gadoxetate disodium (Gd–EOB–DTPA), generally exhibit much lower toxicity, with EC_50_ values exceeding 100 mg/L for most species. This indicates that free gadolinium ions and simple salts are more toxic to aquatic plants compared to its chelated forms [2,75,77,78].

In summary, toxic gadolinium concentrations negatively impact plant health, disrupting growth, nutrient metabolism, and reproduction. These effects are mediated by oxidative stress, nutrient competition, and enzymatic interference. While hormetic effects of gadolinium suggest potential agricultural applications, its toxicity necessitates further research to establish safe thresholds and mitigate environmental risks.

## 5. Impact of Gadolinium on Plant Stress Responses

Abiotic stresses such as drought and salinity significantly hinder plant growth and biomass production. Prolonged exposure to these stresses can cause irreversible cellular damage [80]. High soil salinity reduces water uptake by lowering water potential, while drought induces oxidative stress and disrupts metabolism. Research demonstrates that REEs, including gadolinium, enhance plant resilience to abiotic stresses by activating antioxidant enzymes [81], regulating ion transport, stabilizing proteins and membranes [10], and enhancing the transport of essential nutrients like sodium, potassium, zinc, manganese, and phosphorus, which are vital for photosynthesis and metabolic processes [41,63]. For example, under drought stress, plants synthesize osmotic stress proteins (e.g., heat shock proteins) that stabilize cell membranes, while increased proline and abscisic acid (ABA) levels maintain osmotic balance and limit water loss [3]. Gadolinium applications may enhance these biochemical adaptations, improving tolerance to adverse conditions.

Gadolinium’s ability to enhance drought and salinity resistance has potential applications in agriculture. By improving stress tolerance, gadolinium-treated plants could support food production during challenging climatic conditions. Studies suggest REEs positively influence plant biochemical processes, including enhancing nutrient uptake (i.e., nitrogen, phosphorus, and potassium) and metabolic pathways, increasing nitrate reductase activity, improving nitrogen metabolism, and stimulating the production of phytohormones such as auxins, cytokinins, gibberellins, and ABA [69]. Auxins promote root and shoot growth and cell elongation, cytokinins support cell division, bud development, and delay leaf senescence, and gibberellins are vital in seed germination, stem elongation, and fruit development. Additionally, plants enhance the production of ABA, which activates adaptive mechanisms to unfavorable conditions. ABA, a stress hormone, regulates stomatal closure and seed dormancy. This hormone boost improves chloroplast structure and function, increasing ATP production and glucose essential for development. Increased root biomass and lateral root growth improve nutrient uptake efficiency, leading to higher yields [40]. For example, gadolinium-treated rice showed improved stress defense mechanisms and yield potential [73]. Additionally, gadolinium could enhance the production of secondary metabolites in medicinal plants, boosting levels of isoflavones, flavonoids, glycosides, alkaloids, and terpenes [3]. These metabolites have applications in health and medicinal crop agriculture.

Gadolinium plays a significant role in altering signaling pathways and gene expression, impacting metabolism, stress responses, and, ultimately, plant growth [65,82]. During exposure to gadolinium, plants may regulate the expression of genes encoding metal-binding proteins, such as phytochelatins, and stress-related proteins, including heat shock proteins and antioxidant enzymes [83]. Research on the impact of REEs has confirmed changes in gene activity related to protein processing in the endoplasmic reticulum, phosphate transport, and calcium and iron balance in cells [84,85]. Mechanistically, exposure to gadolinium induces mild oxidative stress due to the overproduction of ROS, which triggers cellular defense mechanisms to neutralize these effects [63]. Antioxidant enzyme production also rises, including superoxide dismutase, peroxidase, and catalase, along with non-enzymatic metabolites like ascorbic acid and glutathione, which protect cells from damage. Additionally, photosynthetic pigments, including carotenoids and xanthophylls, offer protection during oxidative stress. ROS oxidize and degrade chlorophyll *a*, prompting plants to compensate by increasing chlorophyll *b* synthesis. Under stress, plants can convert chlorophyll *b* to chlorophyll *a*, raising the chlorophyll a/b ratio. Enzymes and metabolic processes become activated because there is a reduction in the availability of essential nutrients, so gadolinium acts as a cofactor for enzymes involved in chlorophyll synthesis, photosynthesis, and nitrogen metabolism, thus enhancing chloroplast structure and function [74]. Liu et al. [60] measured total dissolved protein in shoots as a plant health indicator, finding that subtoxic gadolinium concentrations (1.57 and 7.86 mg/L) increased protein levels, while a toxic concentration (31.5 mg/L) led to a significant 24.2% decrease. It was also observed that toxic concentrations led to a decrease in chlorophyll content of about 16.7%, suggesting that the effects of gadolinium compounds are highly concentration-dependent and may vary across different physiological parameters.

Due to oxidative stress, plants may activate processes to limit element transport, preventing damage and forming complexes with organic compounds like phytochelatins and organic acids to reduce toxicity [86]. Han et al. [87] suggested that an increased number of free carboxyl groups in the root cell wall facilitates active metal absorption, while Byrne et al. [88] argued that a pH of 7.0 promotes phosphate precipitation with metals, reducing transport potential. Zhang et al. [73] also found that gadolinium accumulates as phosphates and oxalates in vacuoles, soluble fractions, and the cell walls of roots and shoots. The presence of gadolinium alters the levels of essential elements for transport and assimilation due to competition, with calcium being particularly vulnerable. Gadolinium blocks calcium channels, disrupting cellular signaling and enzyme and protein activities. Excessive oxidative stress from toxic gadolinium concentrations can exceed the plant defense capability, leading to cellular damage and a decrease in biochemical parameters [86].

Gadolinium exhibited greater activity compared to lanthanum. Its exposure caused phosphorus deficiency by inducing phosphate precipitation, which subsequently altered the plant sensitivity to auxins. This change affected gene expression and the proliferation of pericyclic cells [40]. Molecular studies have shown that heavy metals damage genes responsible for chlorophyll content [89]. Demethylation acts as a defense mechanism against plant stress and can serve as a marker of environmental metal content. Epigenetic markers of genomic DNA methylation are sensitive and reliable indicators of genotoxicity. Additionally, studies confirmed gadolinium’s impact at toxic concentrations (7.86–31.5 mg/L) on genomic DNA stability and increased genomic methylation polymorphisms [60].

## 6. Environmental and Ecological Considerations

The availability of gadolinium in the environment poses significant long-term ecological and environmental challenges. When gadolinium enters surface and groundwater, it bioaccumulates in aquatic organisms, such as fish and invertebrates, leading to metabolic disturbances, tissue damage, and even death [20,90]. Reproductive processes in these organisms can also become dysregulated, contributing to population declines. Gadolinium (in REEs mixture) is used in Chinese agriculture as fertilizer and has a negative impact on soil organisms that play an important role in biogeochemical cycles and soil fertility [72]. The balance of soil microorganisms responsible for organic matter decomposition is altered, reducing the availability of essential nutrients [91,92,93]. Moreover, gadolinium interferes with the normal functioning of both aquatic and terrestrial plants. Excessive exposure leads to reduced crop yields owing to the toxic effects of the element by its accumulation in the plant cells. Altered mineral availability in the soil further disrupts normal plant growth, negatively impacting ecosystem stability and productivity [55,94].

### Potential for Bioaccumulation of Gadolinium and Trophic Transfer

Gadolinium exhibits significant potential for bioaccumulation in plant tissues (Table 2) [76,95,96]. Table 2 presents data on the exposure of different plant species to gadolinium compounds. It includes the type and concentration of the compound used, the measured gadolinium content in the dry weight of the plant samples, and the corresponding plant species. Presenting data in the form of exposure and determined concentrations provides greater flexibility, transparency, and comparability, especially in the absence of uniform methods for calculating BCFs. The available literature does not always provide bioaccumulation factors (BCFs), and when such values are reported, they are often expressed in different units. In many cases, the gadolinium concentration in the exposure medium is reported in mass per volume, while the amount accumulated in plants is expressed in mass per dry weight. This lack of consistency prevents the standardization of results to a uniform, dimensionless factor.

**Figure 3:**
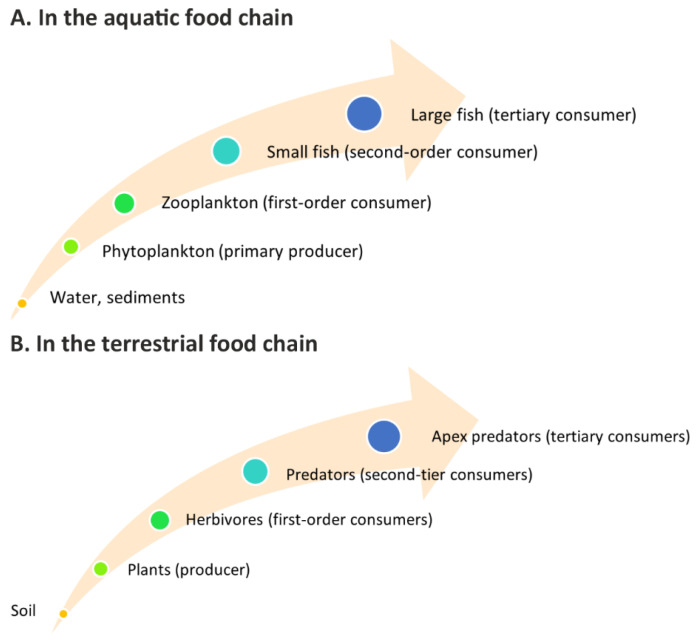
Potential pathways for gadolinium accumulation in the food chain.

The studies of gadolinium bioaccumulation in *Phaeodactylum tricornutum* showed that higher subtoxic concentration of Gd 6.02 mg/L compared to 4.52 mg/L led to higher BCF values of 8.40 and 2.29 in the earlier phases of exposure and showed a decreasing trend with the extension of the experimental time, BCFs being 1.20 and 1.11, respectively, at day 21 [71]. Plants such as *Camelia sinensi* and *Brassica juncea* showed relatively subtoxic Gd concentrations, 0.065–0.7 µg/g and 0.16 µg/g, respectively. In comparison to others, *Capsicum annuum* accumulated higher amounts of gadolinium—0.28 µg/g in roots and 0.13 µg/g in stems [44]. Other review articles also highlight that gadolinium tends to accumulate to a greater extent in roots compared to other plant tissues [60,100,107]. Interestingly, the microalga *Euglena gracilis* demonstrates a lower bioaccumulation potential for gadolinium compared to other REEs like neodymium and erbium [95,108]. The influence of organic ligands on rare earth elements, including gadolinium, has been confirmed through experiments involving ethylenediamine tetraacetic acid (EDTA), nitrilotriacetic acid (NTA), and citrate, which significantly affect bioaccumulation dynamics [18,101]. Studies on contrast agents with linear or macrocyclic structures, both ionic and non-ionic, confirmed the bioaccumulation of gadolinium in *Chlamydomonas reinhardtii* with higher accumulation observed for linear compounds and lower for macrocyclic agents [18]. For Gd-DTPA, concentrations reached 5.6 × 10^−8^ ng/cell in lysis and 1.1 × 10^−8^ ng/cell in sediment. Even higher gadolinium values were found for the Gd-DTPA-BMA compound and amounted to 17 × 10^−8^ ng/cell in sediment. In turn, the Gd-DOTA macrocyclic complex showed significantly lower bioaccumulation as the values were below the limit of quantification (LOD) [18]. Similarly, the maximum concentrations of Gd in *Chlorella kessleri* reached 30 mg/kg for Gd–DOTA, 40 mg/kg for Gd–BOPTA, and 80 mg/kg for Gd(NO_3_)_3_, and the bioconcentration factors were 400, 500, and 1300 L/kg, respectively [43]. In aquatic systems, gadolinium bioaccumulation begins with phytoplankton, which absorbs the element and is subsequently consumed by zooplankton (Figure 3). The element is taken up from water and bottom sediments, where REEs tend to accumulate. Studies have consistently shown that REE concentrations in sediments can be several orders of magnitude higher than those found in the overlying water column, highlighting their pivotal role in the geochemical cycling and availability of these elements within aquatic ecosystems. Furthermore, the strong association of REEs, including gadolinium, with sediment matrices underscores their long-term environmental persistence and potential for trophic transfer through benthic food webs [109].

Bioaccumulation has also been documented in food chain sequences involving *Lemna minor*, daphnia, crustaceans, and goldfish [104]. In this chain (Figure 3A), gadolinium was detected primarily in duckweed and crustaceans, with significant accumulation in phytoplankton. Studies on *Daphnia magna* fed with gadolinium-treated algae confirmed high gadolinium content in the intestines, indicating trophic transfer [61]. The terrestrial food chain begins with plants absorbing gadolinium from the soil (Figure 3B). For instance, the bioaccumulation was observed in *Stevia rebaudiana*, with increasing accumulation amounts (7.88 to 744 µg/g dry weight) corresponding to gadolinium concentrations ranging from 6.050 to 1814 mg/L [74], and in *Arabidopsis thaliana*, exposure to gadolinium resulted in significant root accumulation, with the highest toxicity recorded at 31.5 mg/L concentration [60]. Terrestrial plants are consumed by herbivores. Subsequently, herbivores become prey for predators, which are then consumed by apex predators, ultimately transferring gadolinium to top-level carnivores and humans. This biomagnification process increases the concentration of gadolinium at higher trophic levels, potentially causing physiological and developmental disorders and severe health issues in organisms. Although data on long-term environmental exposure to gadolinium compounds remains limited, ongoing monitoring is essential to mitigate potential risks and prevent adverse effects.

## 7. Strategies for Mitigating Environmental Exposure to Gadolinium

Efforts to minimize the environmental impact of gadolinium require targeted actions and increased public awareness, particularly within the healthcare sector, which is a significant source of gadolinium release. Key strategies include the following: (1) segregation of Post-Contrast Waste by implementing waste segregation protocols in healthcare facilities to prevent gadolinium from entering wastewater systems; (2) recycling and Resource Conservation by collecting gadolinium remnants in designated containers to facilitate recycling and reduce resource wastage; (3) optimized use of GBCA by reducing the administered gadolinium dose by using compounds with higher relaxation efficiency per mmol of gadolinium. Advanced imaging sequences that reduce or eliminate the need for contrast agents should also be prioritized. By adopting these measures, the healthcare sector can play a critical role in mitigating gadolinium’s environmental footprint while conserving this valuable resource for future applications [13,94].

## 8. Future Perspectives

To date, studies of REEs, including gadolinium, have primarily focused on their effects on selected parameters in specific plant species. Due to the chemical similarities among REEs, it is often inferred that their behavior may be comparable across elements. However, detailed studies are required to confirm these assumptions. Currently, limited research has addressed the impact of gadolinium on plants. While gadolinium use has expanded significantly, most studies have been confined to its effects on human exposure and its concentrations in water. Key knowledge gaps include data on plant survival, growth, population dynamics, and reproduction when exposed to varying gadolinium concentrations. For a better understanding of the biological behavior of these elements and their toxicity mechanisms, novel omics approaches should be implemented [110]. Additionally, there is a lack of research on biochemical parameter changes in plants exposed to gadolinium. Metal accumulation in tissues is known to vary over time, making studies on chronic exposure essential. Speciation of gadolinium, which influences its bioavailability and toxicity, is another critical area requiring attention. Moreover, limited information exists regarding the extent of gadolinium accumulation from environmental sources. Comprehensive and detailed studies on specific species are crucial to assess the true environmental impact of gadolinium. Genetic engineering offers significant potential in addressing gadolinium pollution and improving crop resilience. This technology could be applied to crops grown in gadolinium-contaminated soils to enhance resistance. Desired effects could be achieved either by gene modification or by introducing genes encoding proteins that limit gadolinium uptake by plants. For example, increasing the synthesis of phytochelatins, which play a key role in gadolinium detoxification, could improve plant tolerance. Alternatively, traits enabling increased metal accumulation within plant tissues could be developed. Such modifications could involve deactivating genes encoding metal transport proteins, or enhancing the expression of genes importing metals while reducing phytochelatins production [111]. Introducing genes from plants with specific resistance traits is another potential approach. However, any genetic intervention must be preceded by rigorous research to evaluate the environmental implications and avoid unintended consequences [82,112].

In recent years, the types of gadolinium compounds used in medical applications have evolved. In 2017, the European Medicines Agency’s Pharmacovigilance Risk Assessment Committee recommended the withdrawal of linear GBCAs such as gadodiamide, gadopentetate, and gadoversetamide due to evidence of their harmful effects [113]. Similarly, the U.S. Food and Drug Administration issued warnings about the potential side effects of linear gadolinium complexes, advising careful selection of agents for high-risk patients [114].

Research suggests that GBCAs are more harmful than iodine-based compounds. Despite these findings, there remains a lack of comprehensive environmental regulations governing the disposal and management of gadolinium compounds. Developing guidelines for managing gadolinium waste and limiting its use where possible are essential. Moreover, more efficient methods for removing pharmaceutical residues from wastewater should be explored and implemented. Additionally, studies in the literature highlight the potential of recovering gadolinium from patients’ urine, which could serve as a preventive measure to reduce the introduction of gadolinium into the environment [94,115]. Immediate action should focus on reducing gadolinium concentrations in water to prevent further biomagnification through aquatic ecosystems. To address the limitations of current GBCAs, researchers should actively be working on substitutes that maintain high imaging efficacy while minimizing adverse effects on humans and the environment. Promising areas of exploration include the development of biodegradable or less toxic compounds and the use of advanced sequencing methods that could eventually replace contrast agents altogether [116].

## 9. Conclusions

Gadolinium exhibits both beneficial and harmful effects on plants. At subtoxic concentrations, gadolinium can promote plant growth and development by enhancing metabolic and enzymatic activity, improving nutrient absorption, and increasing tolerance to environmental stressors. However, at high concentrations, gadolinium becomes toxic, leading to growth inhibition, oxidative stress, cellular damage, and impaired photosynthesis. This dual nature suggests that gadolinium can function either as a growth-promoting agent or as an inhibitory factor, depending on its concentration and environmental conditions. Despite its growing presence in the environment, there is insufficient data to fully understand how plants respond to gadolinium. The assumption that gadolinium complexes are harmless is concerning, as these complexes can degrade or undergo transmetallation under specific environmental conditions, potentially increasing their bioavailability and toxicity. To better protect the environment, more in-depth research is required to understand the effects of gadolinium on specific plant species and ecosystems.

The industrial and agricultural applications of gadolinium offer several advantages, such as improved technological efficiency and enhanced crop yields. However, these benefits must be weighed against the potential environmental harm, which remains poorly understood. Studies have detected gadolinium in various environmental compartments, including significant concentrations in surface water, groundwater, and even drinking water in multiple regions worldwide. Research has confirmed the accumulation of gadolinium in aquatic organisms and plants, raising concerns about its bioaccumulation and biomagnification potential. Addressing the environmental risks associated with gadolinium will require a coordinated effort among multiple stakeholders, including manufacturers, policymakers, researchers, and consumers. Specific actions include the following: (1) reducing the use of gadolinium by optimizing its application in industry and agriculture to minimize environmental release; (2) segregating gadolinium waste through the implementation of waste management systems to prevent its entry into aquatic ecosystems; (3) monitoring environmental levels by investing in advanced water treatment technologies to remove gadolinium from wastewater and establishing programs to track its presence in ecosystems. Proactive measures by all stakeholders, combined with rigorous monitoring and research, are crucial for mitigating the environmental and ecological impact of gadolinium while preserving its benefits in industrial and agricultural applications.

## Figures and Tables

**Table 1:** Toxicity of gadolinium compounds on plants.

Compound	Plant Species	EC_50_ [mg/L]	References
**Gd^3+^**	*Pseudokirchneriella subcapitata*	2.22	González et al. [77]
	*Lemna gibba*	12.4	Szabó et al. [78]
**GdCl_3_**	*Desmodesmus subspicatus*	4.94	Neubert [79]
**Gd(NO_3_)_3_**	*Skeletonema costatum*	10.2	Tai et al. [2]
	*Raphidocelis subcapitata*	1.21	Joonas et al. [75]
**Gd_2_O_3_**	*Phaeodactylum tricornutum*	6.02	Siciliano et al. [71]
**Gd–DTPA**	*Desmodesmus subspicatus*	>100	Aga et al. [28]
**Gd–DTPA–BMA**		20	Neubert [79]
**Gd–HP–DO3A**		>100	Neubert [79]
**Gd–EOB–DTPA**		>500	Aga et al. [28]
**Gd–BT–DO3A**		937	Aga et al. [28]

**Table 2:** Bioaccumulation of gadolinium compounds in plants.

Compound	Exposure Concentration [mg/L; µg/g *]		Concentration in Dry Weight Sample [µg/g]	Plant Species	References
**Gadolinium**	5.6–8.2 *		0.065–0.07	*Camelia sinensis*	Cao et al. [44]
			0.16	*Brassica juncea*	
			0.28 roots 0.13 stems 0.21 leaves 0.015 fruits	*Capsicum annuum*	
	1.32–7.14 *		0.118–2.87	*Eucalyptus globulus*	Miao et al. [97]
			4.61–32.8	*Dicranopteris dichotoma*	
			0.205–0.734	*Pinus massoniana*	
			0.189	*Phodomyrtus tomentosa*	
			0.296	*Lophatherum gracile*	
			2.52	*Casuarina equisetifolia*	
	2.70–147 *		1.44–85 stems 0.65–3.14 petioles 13.3–76.9 laminas	*Dicranopteris linearis*	Zhenggui et al. [98]
	3.07–6.84 *		0.020–0.065 juice 0.097–0.322 solid	*Vitis vinifera*	Pepi et al. [99]
	3.46–5.33 *		<0.00115 grains 0.00591 stems 0.0305 leaves 0.146 roots	*Zea mays*	Li et al. [100]
			<0.00115 grains 0.018 stems 0.0391 leaves 0.932 roots	*Oryza sativa*	
**Gd^3+^**	0–20		0–1439	*Lemna gibba*	Szabó et al. [78]
	1		6360	*Chlorella vulgaris*	Hao et al. [101]
**GdCl_3_**	3.14		500 × 10^−7^ ng/cell lysate 130 × 10^−6^ ng/cell residue	*Chlamydomonas reinhardtii*	Sommer et al. [18]
**Gd(NO_3_)_3_**	1.57–31.5		67.3–253 roots 2.19–2.73 shoots	*Arabidopsis thaliana*	Liu et al. [60]
	0.1 medium 0.0021–0.00273 MRI 0.00143–0.00273 WWTP		68.3–83.2 medium 4.73–5.22 MRI 3.49–5.23 WWTP	*Chlorella kessleri*	Bendakovská et al. [102]
	10		217	*Zea mays*	Saatz et al. [103]
	0.002–0.1		1–80	*Chlorella kessleri*	Bendakovská et al. [102]
**Gd_2_O_3_**	1.00		104	*Sperollela polyrrhiza*	Yang et al. [104]
	0.01–0.5		14–696	*Ulva lactuca*	Ferreira et al. [105]
	0.01–0.5		17.1–602	*Gracilaria* sp.	
	0.01–0.5		2.40–220	*Fucus spiralis*	
**Gd_2_O_3_ + organic ligands**	1.00	(CIT)	3160	*Chlorella vulgaris*	Hao et al. [101]
		(NTA)	3120		
		(EDTA)	380		
**Gd–DTPA**	5.56		5.6 × 10^−8^ ng/cell lysate 11 × 10^−8^ ng/cell residue	*Chlamydomonas reinhardtii*	Sommer et al. [18]
**Gd–DTPA–BMA**	7.86		9.6 × 10^−8^ ng/cell lysate 17 × 10^−8^ ng/cell residue	*Chlamydomonas reinhardtii*	
	0.256		138	*Ceratophyllum demersum*	Braun et al. [106]
	0.256		39	*Lemna gibba*	
**Gd–DOTA**	5.91		<LOD	*Chlamydomonas reinhardtii*	Sommer et al. [18]
	0.256		39	*Lemna gibba*	Braun et al. [106]
	0.256		59	*Ceratophyllum demersum*	
	1		1–1.1	*Lepidium sativum*	Lindner et al. [107]
	0.002–0.1		0.8–30	*Chlorella kessleri*	Bendakovská et al. [102]
**Gd–BT–DO3A**	7.48		0.59 × 10^−8^ ng/cell lysate 0.29 × 10^−8^ ng/cell residue	*Chlamydomonas reinhardtii*	Sommer et al. [18]
	1		1–1.2	*Lepidium sativum*	Lindner et al. [107]
	6.05–1814		7.88–744	*Stevia rebaudiana*	Scurtu et al. [74]
**Gd–BOPTA**	1		1 stems 2 roots 10 leaves	*Lepidium sativum*	Lindner et al. [107]
	0.002–0.02		1–40	*Chlorella kessleri*	Bendakovská et al. [102]

MRI, Water from magnetic resonance workplace; WWTP, wastewater treatment plant; Gd^3+^, no ligands; CIT, citrate; NTA, nitrilotriacetic acid; EDTA, ethylenediamine tetraacetic acid. * µg/g.

## Data Availability

No new data were created or analyzed in this study.

## Abbreviations

GBCA	gadolinium-based contrast agent
Gd(NO_3_)_3_	gadolinium nitrate
Gd–BOPTA	gadobenate dimeglumine
Gd–BT–DO3A	gadobutrol
GdCl_3_	gadolinium chloride
Gd–DOTA	gadoterate meglumine
Gd–DTPA	gadopentetate dimeglumine
Gd–DTPA–BMA	gadodiamide
Gd–DTPA–BMEA	gadoversetamide
Gd–EOB–DTPA	gadoxetate disodium
Gd–HP–DO3A	gadoteridol
LOD	limit of detection
MRI	magnetic resonance imaging
REE	rare earth element
ROS	reactive oxygen species
WWTP	wastewater treatment plant
